# The effect of the third dose of the BNT162b2 vaccine on anti-SARS-CoV-2 spike antibody levels in healthcare workers with and without COVID-19 infection

**DOI:** 10.1080/07853890.2023.2182907

**Published:** 2023-02-23

**Authors:** Blanka Wolszczak-Biedrzycka, Anna Bieńkowska, Beata Cieślikiewicz, Elwira Smolińska-Fijołek, Grzegorz Biedrzycki, Justyna Dorf

**Affiliations:** a Department of Psychology and Sociology of Health and Public Health, University of Warmia and Mazury in Olsztyn, Olsztyn, Poland; b The Oncology Center of the Region of Warmia and Mazury in Olsztyn, Hospital of the Ministry of the Interior and Administration, Olsztyn, Poland; c Department of Physiology, Medical University of Gdańsk, Gdańsk, Poland; d Hospital Dispensary, Regional Specialist Hospital in Olsztyn, Olsztyn, Poland; e Department of Clinical Laboratory Diagnostics, Medical University of Białystok, Bialystok, Poland

**Keywords:** Third dose of BNT162b2, COVID-19, healthcare workers, anti-SARS-CoV-2S antibodies

## Abstract

**Aim:**

A third (booster) dose of the anti-SARS-CoV-2 vaccine became necessary due to the observed decrease in anti-SARS-CoV-2S antibody levels over time, new mutations, and low global vaccination rates. In this study, anti-SARS-CoV-2S antibody levels were measured (ECLIA assay) in 50 healthcare workers with and without a history of COVID-19 infection to determine the humoral immune response to the third dose of the BNT162b2 vaccine.

**Methods:**

Antibody levels were determined in the blood serum, and blood was sampled for analysis 20–40 days after the administration of the booster dose.

**Results:**

A greater increase in anti-SARS-CoV-2S antibody titers was noted in persons without a history of infection, but antibody levels continued to be higher in previously infected individuals when the results were adjusted for age, gender, BMI, type of work, and presence of comorbidities.

**Conclusion:**

The results of this study can be used to improve the vaccination strategy for the general population.

**KEY MESSAGES**
- Three doses of the vaccine BNT162b2 strongly stimulate the immune system to produce anti-SARS-CoV-2s antibodies, especially in people with a previous infection COVID-19.
- Age, gender, and BMI may be associated with different humoral immune response to the BNT162b2 vaccine.

## 1. Introduction

Vaccination is currently regarded as one of the most effective tools in the fight against the COVID-19 pandemic which has paralyzed the entire world since late 2019. Immunization campaigns against the severe acute respiratory syndrome coronavirus 2 (SARS-CoV-2) began at the end of 2020 in most countries around the world, including Poland, and proved to be highly effective in reducing the transmission and incidence of COVID-19 [1,2].

In most vaccines against COVID-19, including the Pfizer/BioNTech BNT162B2 mRNA vaccine, two doses were required to elicit an immune response and protect against symptoms of infection [3,4]. However, new reports from various countries have shown that the efficacy of all vaccines decreased over time due to new virus mutations and too low global vaccination rates [5,6]. Approximately 55% of the Polish population and <50% of the global population had been fully vaccinated by the end of 2021 [1].

Individual immunity is determined by cellular and humoral immune responses. Research has shown that persons without a history of COVID-19 produced significantly fewer antibodies after vaccination than individuals who had been previously infected [7]. Anti-SARS-CoV-2S antibody levels were also found to decrease over time in fully vaccinated subjects [8]. Antibody titers have been found to remain high several months after vaccination, in particular in persons with a history of COVID-19 [9]. Moreover, infection with SARS-CoV-2 has also been diagnosed in fully vaccinated subjects [10,11]. There are the results of research, which informed that the level of SARS-CoV-2 antibodies is connected with immune protection against COVID-19 [12].

The COVID-19 pandemic has spread around the world at an alarming rate, which is why a booster dose of the vaccine was recommended to strengthen the immune response [13,14]. In Poland, the third dose was initially available only to healthcare workers who are at the greatest risk of becoming infected with SARS-CoV-2. As of 2 November 2021, the booster dose is available to all persons who had completed the full vaccination course at least 6 months earlier [1].

Two mRNA vaccines have been approved for the third dose in Poland: Pfizer/BioNTech BNT162b2 and Moderna mRNA-1273. Both vaccines contain mRNA that encodes the spike (S) protein present on the surface of the SARS-CoV-2 virus which allows the S-protein for binding to the ACE-2 receptor and enter host cells. The efficacy of the Moderna vaccine against COVID-19 had been initially estimated at 94.5%, and the efficacy of the Pfizer/BioNTech vaccine—at 95% [15].

The aim of this study was to determine changes in anti-SARS-CoV-2 antibody levels in healthcare workers 20–40 days after the administration of a third dose of the BNT162B2 vaccine.

## 2. Materials and methods

### 2.1. Materials

The study group consisted of 50 healthcare workers at the Hospital of the Ministry of Internal Affairs and Administration in Olsztyn. The analyzed subjects were aged 25–67, and the sample consisted of both males and females. The participants had been vaccinated with two doses of the BNT162B2 vaccine between 25 January and 17 February 2021 and had received a third dose of the BNT162B2 vaccine between 25 October and 14 November 2021. Antibody levels were determined in all subjects 8 months after the full vaccination course.

The study population was divided into healthcare workers vaccinated with a history of SARS-CoV-2 infection (*n* = 25) confirmed by GeneXpert PCR analysis (based on detection of two target genes: E and N2) before two doses of vaccine (4Q2020), and healthcare workers vaccinated without a history of SARS-CoV-2 infection (*n* = 25) (control the level of IgM/IgG antibodies using rapid cassette tests before vaccination 01.2021).

Demographic data and information about the date of the second and third vaccine dose, side effects, and comorbidities were collected from all participants. Each group was subdivided into subgroups based on gender (male, female), age (≤50, >50), type of work (medical, non-medical), BMI (≤24.9, >24.9), and presence of comorbidities (absent, present). Each participant gave his/her written consent to participate in the study and have blood samples taken.

Blood samples for antibody tests were collected between 1 and 6 December 2021, i.e. 20–40 days after the administration of the third (booster) dose of the BNT162B2 vaccine. Blood was collected into red-top Vacutainer tubes for serum separation by centrifugation. Blood was centrifuged for 10 min at 4000 × g at room temperature. The serum was separated and frozen at −80 °C until analysis.

### 2.2. Method of determining antibody levels

Antibody levels were measured using the Elecsys anti-SARS-CoV-2 S assay (Roche S-RBD tAb). This electrochemiluminescence immunoassay (ECLIA) is used forth *in vitro* quantification of total antibodies (IgG/IgA/IgM) to SARS-CoV-2 S-RBD proteins inhuman serum and is performed on a Roche Cobas E411 fully automated analyzer (Roche Diagnostics). The assay is performed on the Roche Cobas E411(Roche Diagnostics). This assay is a dual antigen assay format using a recombinant protein representing the RBDS antigen. The three-step procedure favors the detection of high-affinity antibodies to SARS-CoV-2. Samples are incubated with a mixture of biotinylated and ruthenylated RBD antigen to create an immune complex with the dual antigen. Streptavidin-coated microparticles are then added to bind the DAGS complexes to the solid phase. The reagent mixture is transferred to a measuring cell, and the microparticles are trapped magnetically. The application of a voltage induce chemiluminescence, which is measured with a photomultiplier tube. The signal output increases as the antibody titer increases. The detection range is 0.40–250 U/ml (up to 25,000 U/ml at a dilution of 1:100), with values below 0.80 U/ml being considered negative and values above 0.80 U/ml being positive [1,16].

### 2.3. Statistical analysis

Statistical data processing was performed using GraphPad Prism 8.4.3 for Windows (GraphPad Software, La Jolla, CA, USA). The distribution of the results was analyzed using Shapiro–Wilk test. Student’s *t*-test was used in case of normal distribution, and Mann–Whitney *U*-test was used to compare data that were not normally distributed. Results are presented as median (min–max). Statistical significance was established at *p* < 0.05.

## 3. Results

### 3.1. Characteristics of the study group

The study included 50 fully vaccinated (after three doses of BNT162b2) hospital workers. The population sample was divided into two groups: subjects with a history of COVID 19 (25 patients) and subjects without a history of COVID 19 (25 patients). In the group with history of COVID-19 only six persons had moderate symptoms of disease, 19 had a mild symptom and nobody was hospitalized. In both groups, the majority of the participants were female (72, 84%), older than 50 (60, 52%), and medical personnel (76, 60%). Detailed characteristics of all groups are presented in Table 1.

**Table 1.** Characteristic of study group.

	Workers with history of COVID-19 (*n* = 25)			*p*-Value	Workers without history of COVID-19 (*n* = 25)			*p*-Value
	Number of workers *n* = 25	Median (5–95% percentile) of anti-SARS-CoV2S level (Uml) (8 months after 2 dose)	Median (5–95% percentile) of anti-SARS-CoV2S level (Uml) (20–40 days after 3 dose)		Number of workers *n* = 25	Median (5–95% percentile) of anti-SARS-CoV2S level (Uml) (8 months after 2 dose)	Median (5–95% percentile) of anti-SARS-CoV2S level (Uml) (20–40 days after 3 dose	
Age								
≤50	15 (60%)	1497 (456.9–8293)	14,725 (1587–25,000)	0.0012	13 (52%)	507.5 (189.8–1707)	13,933 (3246–20,100)	<0.0001
>50	10 (40%)	2564 (901–10,077)	24,323 (21,535–25,000)	0.0007	12 (48%)	522.5 (372.8–770.8)	18,386 (9345–25,000)	<0.0001
Sex								
Female	18 (72%)	4603 (516.5–10,461)	23,271 (11,587–25,000)	<0.0001	21 (84%)	583 (257.5–675)	16,767 (7415–25,000)	<0.0001
Male	7 (28%)	1705 (398–20,229)	20,785 (18,108–23,461)	0.2222	4 (16%)	510 (201.6–1756)	10,334 (3246–18,810)	0.0159
Type of workers								
Medical	19 (76%)	2297 (2559–6183)	18,632 (1587–25,000)	0.0009	15 (60%)	626.0 (210–1891)	15,164 (3246–25,000)	0.0009
Non-medical	6 (24%)	941.5 (542.6–1967)	22,497 (15,082–25,000)	0.0095	10 (40%)	453 (145.7–791.7)	17,134 (7415–25,000)	0.0095
BMI								
≤24.9	7 (28%)	1750 (661–25,000)	18,990 (14,367–25,000)	0.0017	11 (44%)	525 (217–1755)	13,895 (3246–20,100)	<0.0001
>24.9	18 (72%)	3189 (901–24,726)	18,553 (1587–25,000)	0.0139	14 (56%)	581 (225–1564)	18,523 (9345–25,000)	<0.0001
Other diseases								
Present	19 (76%)	2780 (686.2–24,918)	15,082 (14,367–25,000)	0.0070	10 (40%)	640 (235–1564)	15,164 (7415–25,000)	0.0070
Absent	6 (24%)	2090 (1320–6987)	16,325 (8108–25,000)	<0.0001	15 (60%)	559 (217–1755)	16,325 (3246–24,626)	<0.0001

### 3.2. Comparison of total anti-SARS-CoV-2 antibodies level depending on the sex, age, BMI, coexisting diseases, and work type in group of workers with history of COVID 19

**Figure 1.**
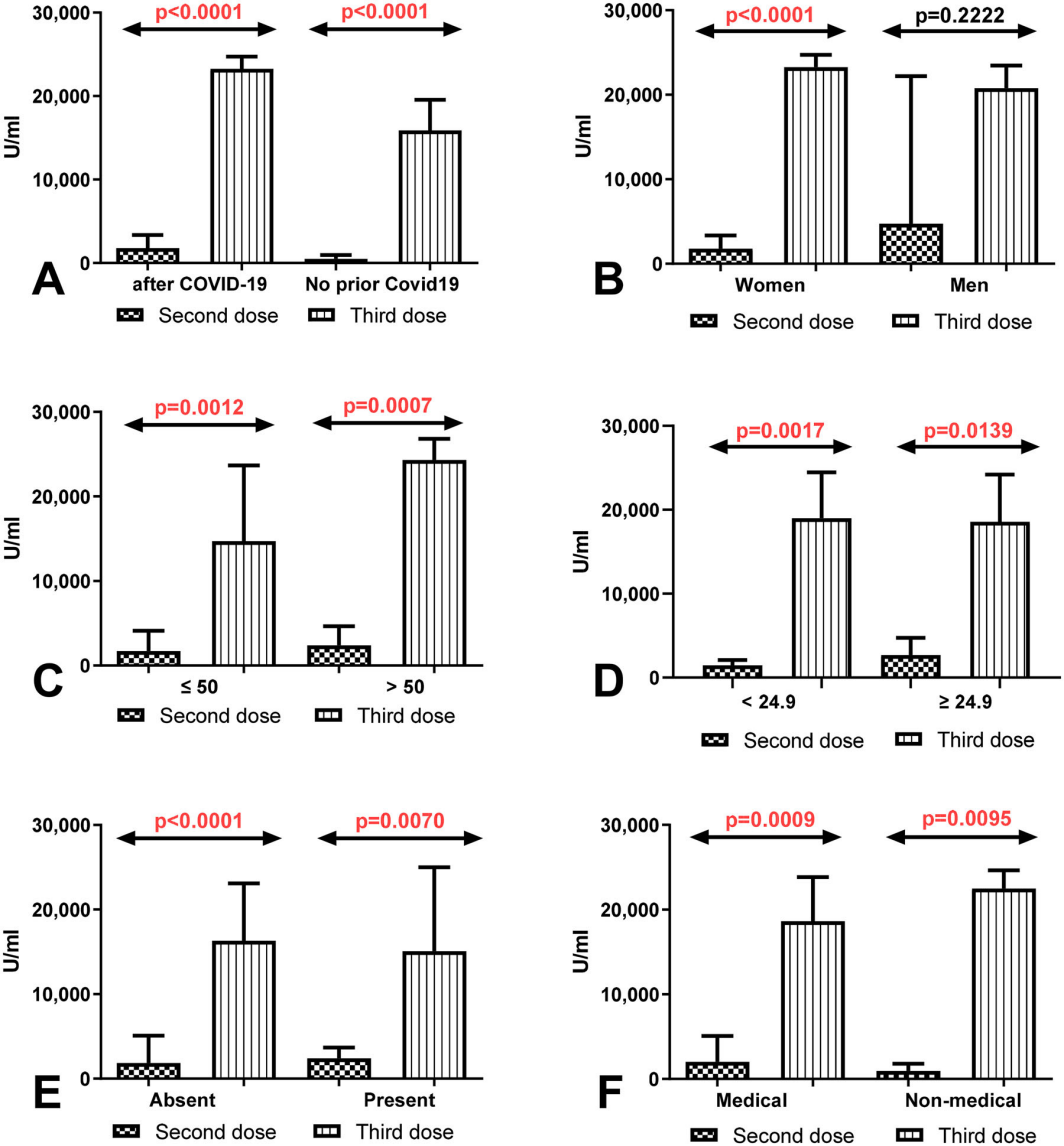
(A) Comparison of total anti-SARS-CoV-2 antibodies level in the groups of workers with and without a history of COVID19 previously vaccinated with a 2-dose and 3-dose series of BNT162b2. The data are presented as median (interquartile range). (B) Comparison of total anti-SARS-CoV-2 antibodies level in the groups of women and men with a history of COVID19 previously vaccinated with a 2-dose and 3-dose series of BNT162b2. The data are presented as median (interquartile range). (C) Comparison of total anti-SARS-CoV-2 antibodies level in the groups of workers before and after the age of 50 with a history of COVID19 previously vaccinated with a 2-dose and 3-dose series of BNT162b2. The data are presented as median (interquartile range). (D) Comparison of total anti-SARS-CoV-2 antibodies level in the group of workers with normal and increased BMI with a history of COVID19 previously vaccinated with a 2-dose and 3-dose series of BNT162b2. The data are presented as median (interquartile range). (E) Comparison of total anti-SARS-CoV-2 antibodies level in the groups of workers with and without coexisting diseases with a history of COVID19 previously vaccinated with a 2-dose and 3-dose series of BNT162b2. The data are presented as median (interquartile range). (F) Comparison of total anti-SARS-CoV-2 antibodies level in the groups of medical and non-medical workers with a history of COVID19 previously vaccinated with a 2-dose and 3-dose series of BNT162b2. The data are presented as median (interquartile range).

The result showed statistically significant increase in total anti-SARS-CoV-2S antibodies level in the group of workers with history of Covid19 (*p* < 0.0001) and without a history of Covid19 (*p* < 0.0001) after immunization of the third dose compared to the total anti-SARS-CoV-2 antibodies level measured after 8 months after immunization of second dose (Figure 1(A)). In the group of women with a history of Covid19, the level of total anti-SARS-CoV-2 antibodies after the third dose was considerably higher compared to its level 8 months of receiving the second dose (*p* < 0.0001) (Figure 1(B)). Similarly in the group of workers before and after 50 age as well as workers with normal and higher BMI factor with a history of Covid19 the level of total anti-SARS-CoV-2 antibodies was significantly higher after the third dose in comparison with its level after 8 months of receiving second dose (Figures 1(C,D)). Total anti-SARS-CoV-2 antibodies level was also higher in workers with and without coexisting diseases and in the groups of medical and non-medical workers with a history of Covid19 after receiving the third dose than in the same groups after 8 months of the second dose (Figure 1(E,F)).

### 3.3. Comparison of total anti-SARS-CoV-2 antibodies level depending on the sex, age, BMI, coexisting diseases, and work type in group of workers without history of COVID 19

**Figure 2.**
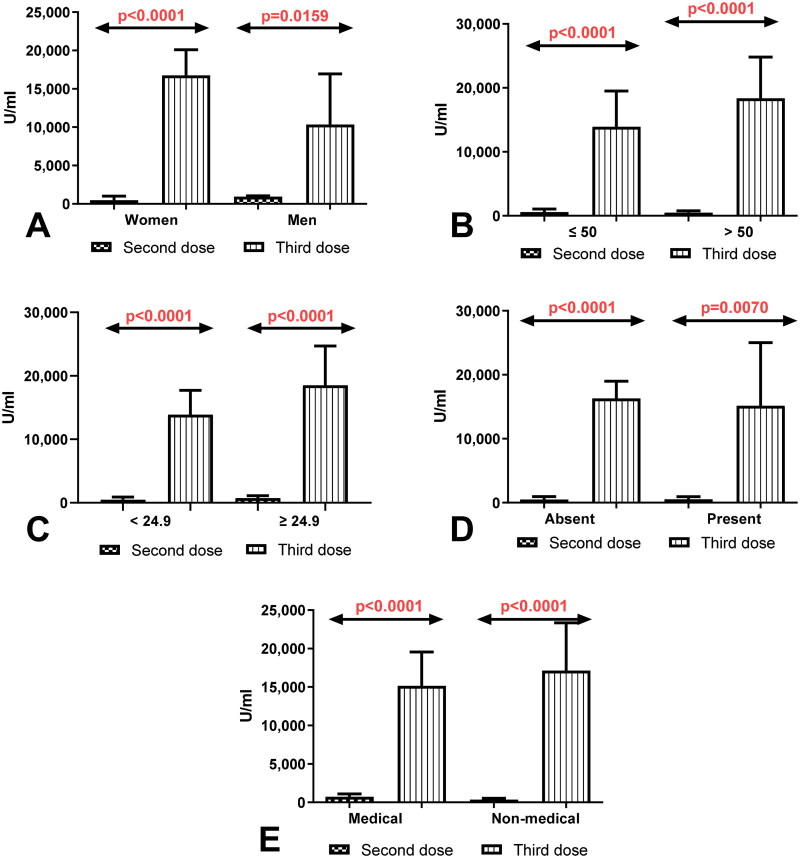
(A) Comparison of total anti-SARS-CoV-2 antibodies level in the groups of women and men without a history of COVID19 previously vaccinated with a 2-dose and 3-dose series of BNT162b2. The data are presented as median (interquartile range). (B) Comparison of total anti-SARS-CoV-2 antibodies level in the groups of patients before and after the age of 50 without a history of COVID19 previously vaccinated with a 2-dose and 3-dose series of BNT162b2. The data are presented as median (interquartile range). (C) Comparison of total anti-SARS-CoV-2 anti-bodies level in the group of patients with normal and increased BMI without a history of COVID19 previously vaccinated with a 2-dose and 3-dose series of BNT162b2. The data are presented as median (interquartile range). (D) Comparison of total anti-SARS-CoV-2 antibodies level in the groups of patients with and without coexisting diseases without a history of COVID19 previously vaccinated with a 2-dose and 3-dose series of BNT162b2. The data are presented as median (interquartile range). The data are presented as median (interquartile range). (E) Comparison of total anti-SARS-CoV-2 antibodies level in the groups of medical and non-medical workers without a history of COVID19 previously vaccinated with a 2-dose and 3-dose series of BNT162b2. The data are presented as median (interquartile range).

Considerably higher level of total anti-SARS-CoV-2 antibodies in the group of women (*p* < 0.0001) and men (*p* = 0.0230) without history of COVID19 after having a third dose compared to the total anti-SARS-CoV-2 antibodies level determined after 8 months of receiving second dose were observed (Figure 2(A)). Simultaneously, the level of total anti-SARS-CoV-2 antibodies was higher in workers before 50 (*p* < 0.0001) and after 50 years old (*p* < 0.0001) and also in the group of workers with normal (*p* < 0.0001) and higher (*p* < 0.0001) BMI factor without a history of COVID19 after the third dose of vaccination compared to the level of antibodies measured 8 months after having second dose (Figures 2(B,C)). The total anti-SARS-CoV-2 antibodies level after the third dose of vaccination was higher in workers with and without coexisting diseases and in the groups of medical and non-medical workers without history of Covid19 after receiving the third dose than in the same groups after 8 months of the second dose (Figures 2(D,E)).

### 3.4. Comparison of total anti-SARS-CoV-2 antibodies level depending on the sex, age, BMI, coexisting diseases, and work type between group of workers with and without history of COVID 19 after having third dose of vaccine

**Figure 3.**
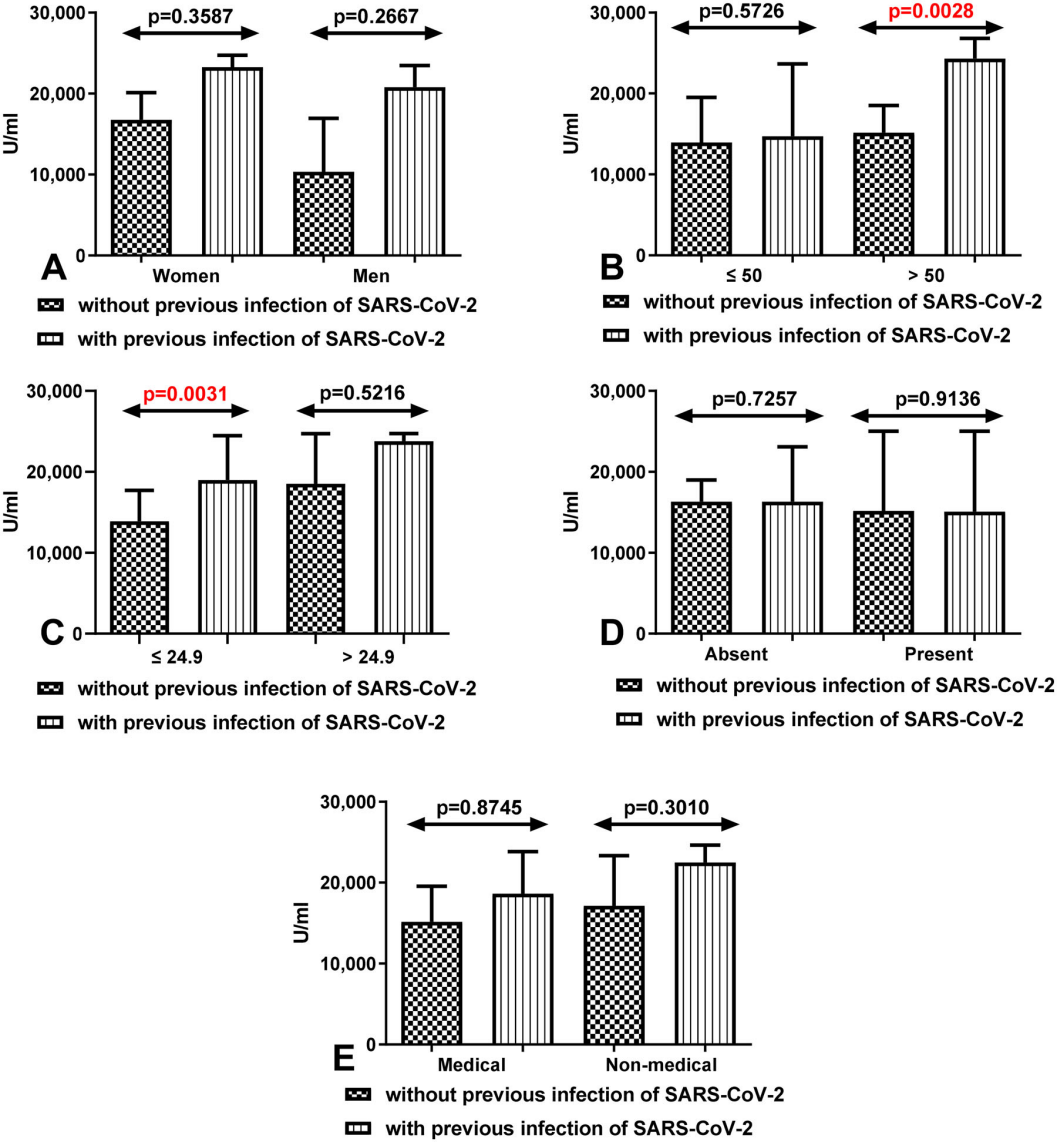
(A) Comparison of total anti-SARS-CoV-2 antibodies level between group of women and men without and with a history of COVID-19 after having a third dose of vaccine. The data are presented as median (interquartile range). (B) Comparison of total anti-SARS-CoV-2 antibodies level between groups of patients before and after the age of 50 without and with a history of COVID-19 after having a third dose of vaccine. The data are presented as median (interquartile range). (C) Comparison of total anti-SARS-CoV-2 antibodies level in the group of workers with normal and increased BMI without and with a history of COVID19 after having a third dose of vaccine. The data are presented as median (interquartile range). (D) Comparison of total anti-SARS-CoV-2 antibodies level in the groups of workers with and without coexisting diseases without and with a history of COVID19 after having a third dose of vaccine. The data are presented as median (interquartile range). The data are presented as median (interquartile range). (E) Comparison of total anti-SARS-CoV-2 antibodies level in the groups of medical and non-medical workers without and with a history of COVID19 after having a third dose of vaccine. The data are presented as median (interquartile range).

We also demonstrated that total anti-SARS-CoV-2S antibodies level was increased in all compared group of workers with Covid19 than in workers without Covid19. Statistically significant differences between both group of workers were especially seen in people after 50 years old with Covid19 compared to those without Covid19 (*p* = 0.0028) (Figure 3(B)). In the group of workers with and without Covid19 with normal BMI the differences were also statistically significant (*p* = 0.0031) (Figure 3(C)).

## 4. Discussion

Numerous researchers have determined antibody levels after the administration of different vaccine brands, within different periods of time after the first and the second dose, in persons with and without a history of COVID-19, in subjects with comorbidities, and in individuals from different demographic groups [7,17–20]. The results of published studies indicate that antibody levels remain high several months after the administration of two vaccine doses [21–23]. This is a highly satisfactory outcome, but COVID-19 infections have also been diagnosed in vaccinated subjects [11]. Therefore, vaccination schedules had to be modified, and a booster dose was introduced to stimulate humoral and cellular immune responses against the virus [24].

Due to the fact that the correlates of protection against SARS-CoV-2 and protective titers have not been defined yet, it is difficult to determine whether a booster dose is necessary for the general population. Nonetheless, various demographic groups should be studied to analyze the level of immunity conferred by each dose of the approved vaccine brands [25]. The existing research focused mainly on humoral and cellular responses to the third dose of the BNT162B2 vaccine in priority patients, i.e. seniors, persons with comorbidities and immunocompromised patients [26,27]. In the present study, the immune response after the administration of the third dose of the Pfizer/BioNTech vaccine was evaluated in healthcare workers aged 25–67. Around one month after the administration of the third dose, anti-SARS-CoV-2S antibody levels increased more than 10-fold in subjects with a history of COVID-19 infection relative to the levels determined eight months after the second dose (median increase from 2162 to 23,271 U/ml), whereas, in individuals without a history of COVID-19 infection, anti-SARS-CoV-2S antibody levels increased more than 30-fold (median increase from 522 to 15,882 U/ml). Despite a much smaller increase in antibody levels, persons with a history of COVID-19 continued to have significantly higher levels of anti-SARS-CoV-2S antibodies (*p* < 0.0001), which could suggest that contracting the coronavirus is as effective as a single vaccine dose [28]. Similar observations were made by Munro et al. in a blinded, multicenter, randomized, controlled phase II clinical trial. Their results were published in *Lancet*, and they revealed that antibody titers after the third dose of both BNT162b2 and ChAdOx1 (AstraZeneca) vaccines were higher in persons with a history of COVID-19 than in subjects who had not been previously infected with the SARS-CoV-2 virus. However, the noted differences were smaller than those observed after the second dose, which is consistent with our findings [29].

The humoral immune response may be affected by age. Gilboa et al. [26] reported that neutralizing antibody levels increased more than 9-fold in healthcare workers older than 60. In the current study, a booster dose of the BNT162B2 vaccine increased neutralizing antibody levels more than 35 times in 50+ healthcare workers without a history of COVID-19 and more than nine times in 50+ healthcare workers with a history of infection. It should be emphasized that mean anti-SARS-CoV-2S antibody titers were significantly higher (*p* < 0.05) in subjects older than 50 than in individuals aged ≤50.

In successive months of the pandemic, researchers observed that new variants of SARS-CoV-2 could weaken natural immunity as well as vaccine-induced immunity. The B.1.617.2 (Delta) variant of SARS-CoV-2 is responsible for the recent increase in the incidence of COVID-19 infections around the world. A recent study demonstrated only minor differences in the efficacy of two doses of the BNT162b2 vaccine against the Delta and Alpha variants. The efficacy of two BNT162b2 doses was determined at 93.7% in patients infected with the Alpha variant and 88.0% in individuals infected with the Delta variant [30]. A study analyzing neutralizing antibodies against the original SARS-CoV-2 strain identified in Wuhan (WA1/2020) and the B.1.617.2 (Delta) variant revealed that the B.1.617.2 variant was 2.9 times less susceptible to neutralization by the serum of vaccinated individuals than the WA1/2020 variant. However, all serum samples from vaccinated subjects were characterized by detectable neutralizing activity against the Delta variant [31]. These findings suggest that a third dose of the BNT162B2 vaccine effectively addresses waning immunity over time.

The booster dose can also produce adverse side effects. Some studies reported that the side effects of the third dose were similar (mild to severe) to those observed after the first and the second dose [31,32], whereas other researchers [33] found that side effects were mild and different from those noted after the first two doses. In the present study, only mild side effects were noted after the booster dose, and none of them lasted more than 24 h. In contrast, the side effects associated with the second dose were described by some participants as moderate to severe.

Before the third dose was introduced, attempts had been made to determine which population group would benefit most from the booster dose and should receive priority. Special attention was paid to immunocompromised subjects who may not have developed sufficient immunity after the first two doses of the vaccine. Kamar et al. [34] studied immunocompromised patients (recipients of solid organ transplants) and detected anti-SARS-CoV-2 antibodies in only 40% of the subjects (40 out of 99) four weeks after the administration of the second dose. However, antibodies were detected in 68% of the examined patients (67 out of 99) four weeks after the third dose. Similar observations were made by Werbel et al. [35] in a study of 30 recipients of solid organ transplants. Antibody levels increased after the third dose (24–101 days after the second dose) in one-third of the patients (*n* = 6) who had not developed anti-SARS-CoV-2 antibodies after the first two doses (*n* = 24) and in all patients with low antibody levels (*n* = 6). Peled et al. [33] examined immune responses to a booster dose in heart transplant patients. A humoral immune response was noted in only 23% of the subjects after two vaccines, and anti-SARS-CoV-2 antibodies were detected in 67% of the subjects after the third dose. In other studies, antibody levels also increased in dialysis patients after a third dose of the Pfizer/BioNTech vaccine, and the highest increase was noted in patients who had responded weakly to the first two doses of the vaccine [36].

These results indicate that a booster dose is effective in patients who are at the highest risk of infection with SARS-COV-2S. The potential impact of pre-existing comorbidities on antibody levels was also analyzed in the present study. However, no significant differences were found between subjects with comorbidities (diabetes, cardiovascular diseases, hematological diseases, kidney diseases, autoimmune diseases) and potentially healthy individuals.

As previously mentioned, vaccination produces a stronger humoral immune response in persons with a history of COVID-19 infection, which indicates that the vaccination schedule can be modified and that a single model should not be applied to the general population. Clinical trials on smaller booster doses of the BNT162b2 vaccine (5 or 10 µg) than those administered to healthy adults (30 µg) are currently under way [37]. Attempts are also being made to develop a prototype vaccine (30 µg dose) against the South African variant of the virus [38].

The current study examined the humoral immune response to a third dose of the same vaccine (BNT162b2). However, the efficacy of different COVID-19 vaccines as boosters has also been investigated. Munro et al. examined seven vaccines that could be administered as boosters after two doses of BNT162b2 or ChAdOx1. All vaccines produced satisfactory humoral immune responses [29]. Soytas et al. demonstrated that the use of BNT162b2 as a booster after two doses of ChAdOx1 induced a stronger humoral immune response than three ChAdOx1 doses [39]. The administration of BNT162b2 as a booster after two doses of CoronaVac was also recommended by Turkish researchers [40]. Interesting results were reported by Faustini et al. who found that the administration of BNT162b2 as a booster after two doses of ChAdOx1 offered greater protection against the Delta variant that three doses of BNT162b2. However, subjects who received three doses of BNT162b2 developed greater immunity against the Omicron variant [41,42].

Vaccines against COVID-19 have immense potential to confer long-lasting humoral immunity. However, further research is needed to define the correlates of immunity and protective titers to determine the need for the third and successive booster doses. The protection conferred by the booster dose should be analyzed in the context of global vaccine shortages to identify the most susceptible populations without compromising the global vaccination campaign against COVID-19.

## 5. Conclusions

This study demonstrated that a booster dose of the BNT162b2 vaccine produced a strong humoral immune response in healthcare workers. Antibody titers were significantly higher in fully vaccinated subjects with a history of COVID-19 infection, which could be attributed to the development of natural immunity. However, after the booster dose, the increase in antibody levels was lower in persons with a history of COVID-19 than in subjects who had not been infected with SARS-CoV-2. The present findings can be used to optimize the vaccination program.

## Data Availability

The full data presented in this study are available on request from the corresponding author.

## References

[1] [cited 2021 Jun 10]. Available from: http://www.gov.pl/>web>szczepimysie

[2] Harvey WT, Carabelli AM, Jackson B, et al. SARS-CoV-2 variants, spike mutations and immune escape. Nat Rev Microbiol. 2021;19:409–424. 34075212 10.1038/s41579-021-00573-0 PMC8167834

[3] al Kaabi N, Zhang Y, Xia S, et al. Effect of 2 inactivated SARS-CoV-2 vaccines on symptomatic COVID-19 infection in adults: a randomized clinical trial. JAMA. 2021;326:35–45. 34037666 10.1001/jama.2021.8565 PMC8156175

[4] Zhang Y, Zeng G, Pan H, et al. Safety, tolerability, and immunogenicity of an inactivated SARS-CoV-2 vaccine in healthy adults aged 18–59 years: a randomised, double-blind, placebo-controlled, phase 1/2 clinical trial. Lancet Infect Dis. 2021;21:181–192. 33217362 10.1016/S1473-3099(20)30843-4 PMC7832443

[5] Goldberg Y, Mandel M, Bar-On YM, et al. Waning immunity after the BNT162b2 vaccine in Israel. N Engl J Med. 2021;385:e85. 34706170 10.1056/NEJMoa2114228 PMC8609604

[6] Levin EG, Lustig Y, Cohen C, et al. Waning immune humoral response to BNT162b2 Covid-19 vaccine over 6 months.N Engl J Med. 2021;385:e84. 34614326 10.1056/NEJMoa2114583 PMC8522797

[7] Salvagno GL, Henry BM, Pighi L, et al. Three-month analysis of total humoral response to Pfizer BNT162b2 mRNA COVID-19 vaccination in healthcare workers. J Infect. 2021;83:e4–e5. 10.1016/j.jinf.2021.06.024 PMC8241575 34214516

[8] Israel A, Merzon E, Schäffer AA, et al. Elapsed time since BNT162b2 vaccine and risk of SARS-CoV-2 infection in a large cohort. medRxiv. 2021:2021.08.03.21261496. Available from: 10.1101/2021.08.03.21261496 PMC9083235 34819275

[9] Tartof SY, Slezak JM, Fischer H, et al. Effectiveness of mRNA BNT162b2 COVID-19 vaccine up to 6 months in a large integrated health system in the USA: a retrospective cohort study. Lancet. 2021;398:1407–1416. 34619098 10.1016/S0140-6736(21)02183-8 PMC8489881

[10] Bergwerk M, Gonen T, Lustig Y, et al. Covid-19 breakthrough infections in vaccinated health care workers.N Engl J Med. 2021;385:1474–1484. 34320281 10.1056/NEJMoa2109072 PMC8362591

[11] Angel Y, Spitzer A, Henig O, et al. Association between vaccination with BNT162b2 and incidence of symptomatic and asymptomatic SARS-CoV-2 infections among health care workers. JAMA. 2021;325:2457–2465. 33956048 10.1001/jama.2021.7152 PMC8220476

[12] Khoury DS, Cromer D, Reynaldi A, et al. Neutralizing antibody levels are highly predictive of immune protection from symptomatic SARS-CoV-2 infection. Nat Med. 2021;27:1205–1211. 34002089 10.1038/s41591-021-01377-8

[13] Pouwels KB, Pritchard E, Matthews PC, et al. Effect of delta variant on viral burden and vaccine effectiveness against new SARS-CoV-2 infections in the UK. Nat Med. 2021;27:2127–2135. 34650248 10.1038/s41591-021-01548-7 PMC8674129

[14] Barda N, Dagan N, Cohen C, et al. Effectiveness of a third dose of the BNT162b2 mRNA COVID-19 vaccine for preventing severe outcomes in Israel: an observational study. Lancet. 2021;398:2093–2100. 34756184 10.1016/S0140-6736(21)02249-2 PMC8555967

[15] Thompson MG, Burgess JL, Naleway AL, et al. Prevention and attenuation of Covid-19 with the BNT162b2 and mRNA-1273 vaccines. N Engl J Med. 2021;385:320–329. 34192428 10.1056/NEJMoa2107058 PMC8262622

[16] Voysey M, Costa Clemens SA, Madhi SA, et al. Single-dose administration and the influence of the timing of the booster dose on immunogenicity and efficacy of ChAdOx1 nCoV-19 (AZD1222) vaccine: a pooled analysis of four randomised trials. Lancet. 2021;397:881–891. 33617777 10.1016/S0140-6736(21)00432-3 PMC7894131

[17] Gobbi F, Buonfrate D, Moro L, et al. Antibody response to the BNT162b2 mRNA COVID-19 vaccine in subjects with prior SARS-CoV-2 infection. Viruses. 2021;13:1–10. 10.3390/v13030422 PMC8001674 33807957

[18] Subbarao S, Warrener LA, Hoschler K, et al. Robust antibody responses in 70–80-year-olds 3 weeks after the first or second doses of Pfizer/BioNTech COVID-19 vaccine, United Kingdom, January to February 2021. Eurosurveillance. 2021;26:1–6. 10.2807/1560-7917.ES.2021.26.12.2100329 PMC7995559 33769252

[19] Zhong D, Xiao S, Debes AK, et al. Durability of antibody levels after vaccination with mRNA SARS-CoV-2 vaccine in individuals with or without prior infection. J Am Med Assoc. 2021;326:1–8. 10.1001/jama.2021.19996 PMC8561429 34724529

[20] Wolszczak-Biedrzycka B, Bieńkowska A, Zaborowska JE, et al. Anti-SARS-CoV-2S antibody levels in healthcare workers 10 months after the administration of two BNT162b2 vaccine doses in view of demographic characteristic and previous COVID-19 infection. Vaccines. 2022;10:1–10. 10.3390/vaccines10050741 PMC9146273 35632498

[21] Campo F, Venuti A, Pimpinelli F, et al. Antibody persistence 6 months post-vaccination with BNT162b2 among health care workers. Vaccines. 2021;9:14–22. 10.3390/vaccines9101125 PMC8538824 34696233

[22] Doria-Rose N, Suthar MS, Makowski M, et al. Antibody persistence through 6 months after the second dose of mRNA-1273 vaccine for Covid-19. N Engl J Med. 2021;384:2259–2261. 33822494 10.1056/NEJMc2103916 PMC8524784

[23] Wolszczak-Biedrzycka B, Bié Nkowska A, Dorf J, et al. Assessment of post-vaccination antibody response eight months after the administration of BNT1622b2 vaccine to healthcare workers with particular emphasis on the impact of previous COVID-19 infection. Vaccines. 2021;9:1508. 34960254 10.3390/vaccines9121508 PMC8704861

[24] Smoot K, Yang J, Tacker DH, et al. Persistence and protective potential of SARS-CoV-2 antibody levels after COVID-19 vaccination in a West Virginia Nursing Home Cohort. JAMA Netw Open. 2022;5:e2231334. 36098966 10.1001/jamanetworkopen.2022.31334 PMC9471977

[25] Shekhar R, Garg I, Pal S, et al. COVID-19 vaccine booster: to boost or not to boost. Infect Dis Rep. 2021;13:924–929. 34842753 10.3390/idr13040084 PMC8628913

[26] Gilboa M, Mandelboim M, Indenbaum V, et al. Early immunogenicity and safety of the third dose of BNT162b2 mRNA Covid-19 vaccine among adults older than 60 years; real world experience. J Infect Dis. 2021;5:1–10. 10.1093/infdis/jiab584 34850049

[27] Peled Y, Ram E, Lavee J, et al. Third dose of the BNT162b2 vaccine in heart transplant recipients: immunogenicity and clinical experience. J Heart Lung Transplant. 2021;5:9–18. 10.1016/j.healun.2021.08.010 PMC8397500 34565682

[28] Krammer F, Srivastava K, Alshammary H, et al. Antibody responses in seropositive persons after a single dose of SARS-CoV-2 mRNA vaccine. N Engl J Med. 2021;384:1372–1374. 33691060 10.1056/NEJMc2101667 PMC8008743

[29] Munro APS, Janani L, Cornelius V, et al. Safety and immunogenicity of seven COVID-19 vaccines as a third dose (booster) following two doses of ChAdOx1 nCov-19 or BNT162b2 in the UK (COV-BOOST): a blinded, multicentre, randomised, controlled, phase 2 trial. Lancet. 2021;398:2258–2276. 34863358 10.1016/S0140-6736(21)02717-3 PMC8639161

[30] Bernal JL, Andrews N, Gower C, et al. Effectiveness of COVID-19 vaccines against the B.1.617.2 variant. medRxiv. 2021:2021.05.22.21257658. Available from: 10.1101/2021.05.22.21257658

[31] Falsey AR, Frenck RW, Walsh EE, et al. SARS-CoV-2 neutralization with BNT162b2 vaccine dose 3. N Engl J Med. 2021;385:1627–1629. 34525276 10.1056/NEJMc2113468 PMC8461567

[32] Hall VG, Ferreira VH, Ku T, et al. Randomized trial of a third dose of mRNA-1273 vaccine in transplant recipients. N Engl J Med. 2021;385:1244–1246. 34379917 10.1056/NEJMc2111462 PMC8385563

[33] Peled Y, Ram E, Lavee J, et al. Third dose of the BNT162b2 vaccine in heart transplant recipients: immunogenicity and clinical experience. J Heart Lung Transplant. 2021;40:759–762. 34565682 10.1016/j.healun.2021.08.010 PMC8397500

[34] Kamar N, Abravanel F, Marion O, et al. Three doses of an mRNA Covid-19 vaccine in solid-organ transplant recipients. N Engl J Med. 2021;385:661–662. 34161700 10.1056/NEJMc2108861 PMC8262620

[35] Werbel WA, Boyarsky BJ, Ou MT, et al. Safety and immunogenicity of a third dose of SARS-CoV-2 vaccine in solid organ transplant recipients: a case series. Ann Intern Med. 2021;174:1330–1332. 34125572 10.7326/L21-0282 PMC8252023

[36] Bensouna I, Caudwell V, Kubab S, et al. SARS-CoV-2 antibody response after a third dose of the BNT162b2 vaccine in patients receiving maintenance hemodialysis or peritoneal dialysis. Am J Kidney Dis. 2021;10:1–11. 10.1053/j.ajkd.2021.08.005 PMC8425695 34508833

[37] Study to describe the safety, tolerability, immunogenicity, and efficacy of RNA vaccine candidates against COVID-19 in healthy individuals – Full text view – ClinicalTrials.gov [Internet]. [cited 2022 Jan 11]. Available from: https://clinicaltrials.gov/ct2/show/NCT04368728

[38] Flaxman A, Marchevsky NG, Jenkin D, et al. Reactogenicity and immunogenicity after a late second dose or a third dose of ChAdOx1 nCoV-19 in the UK: a substudy of two randomised controlled trials (COV001 and COV002). Lancet. 2021;398:981–990. 34480858 10.1016/S0140-6736(21)01699-8 PMC8409975

[39] Soytas RB, Cengiz M, Islamoglu MS, et al. Antibody responses to COVID-19 vaccines in older adults. J Med Virol. 2021;6:1–15. 10.1002/jmv.27531 34921432

[40] Keskin AU, Bolukcu S, Ciragil P, et al. SARS-CoV-2 specific antibody responses after third CoronaVac or BNT162b2 vaccine following two-dose CoronaVac vaccine regimen. J Med Virol. 2022;94:39–41. 34536028 10.1002/jmv.27350

[41] Faustini SE, Shields AM, Banham G, et al. Cross reactivity of spike glycoprotein induced antibody against delta and omicron variants before and after third SARS-CoV-2 vaccine dose. medRxiv. 2022:2021.12.30.21268308. Available from: 10.1101/2021.12.30.21268308 PMC8743815 35016901

[42] Higgins V, Fabros A, Kulasingam V. Quantitative measurement of anti-SARS-CoV-2 antibodies: analytical and clinical evaluation. J Clin Microbiol. 2021;59:1–14. 10.1128/JCM.03149-20 PMC8092751 33483360

